# Exploring the Anticancer, Anti‐Inflammatory, and Antibiofilm Efficacy of Endemic *Stachys longiflora*: Insights into the Phytochemical Composition

**DOI:** 10.1002/open.202500146

**Published:** 2025-07-20

**Authors:** Ecem GÜLDAR, Sevim Feyza ERDOĞMUŞ, Cengiz SARIKURKCU

**Affiliations:** ^1^ Department of Basic Pharmaceutical Sciences Faculty of Pharmacy Afyonkarahisar Health Sciences University Afyonkarahisar 03030 Turkey; ^2^ Department of Pharmaceutical Microbiology Faculty of Pharmacy Afyonkarahisar Health Sciences University Afyonkarahisar 03030 Turkey; ^3^ Department of Analytical Chemistry Faculty of Pharmacy Afyonkarahisar Health Sciences University Afyonkarahisar 03030 Turkey

**Keywords:** antibiofilms, anticancer, anti‐inflammatory, antioxidants, *Stachys longiflora*

## Abstract

This study investigates the phytochemical profile, antibiofilm, and anticancer properties of the endemic *Stachys longiflora*. Phytochemical analysis is performed using liquid chromatography coupled with electrospray ionization tandem mass spectrometry, identifying 19 compounds, with verbascoside as the major component (12 290 ± 21 μg g^−1^). The anticancer activity of the extract is assessed on MCF‐7 breast cancer cells using the MTT assay. IC_50_ value is determined to be 5 mg mL^−1^. Oxidative stress parameters, including total oxidant and antioxidant status, along with inflammatory cytokine levels, are measured in cell lysates. The cytokines TNF‐α, TGF‐β, DEF‐β, and IL‐1β are found to be elevated in all treatment groups compared to the control. Antibiofilm activity against *Staphylococcus aureus* ATCC 25923 is evaluated using the MTT method and scanning electron microscopy. The minimum inhibitory concentration of the plant extract is determined to be 250 μg mL^−1^. Biofilm inhibition and biofilm eradication increase in parallel with the concentration of the plant extract. At 2× MIC, biofilm inhibition and eradication are 74.37 ± 1.39% and 44.99 ± 1.03%, respectively. These findings highlight *Stachys longiflora* as a promising source of bioactive compounds with significant antibiofilm and anticancer properties.

## Graphical abstract

*Stachys longiflora* extract was prepared by ultrasonic extraction method. The phytochemicals in the extracts were analyzed by liquid chromatography‐electrospray tandem mass spectrometry. The antibiofilm activity against *Staphylococcus aureus* ATCC 25923 was assessed using the MTT method and scanning electron microscopy. Also, the anticancer activity of the plant extract was evaluated on MCF‐7 cells.

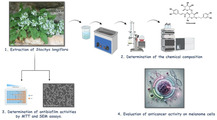

## 1 Introduction

Cancers are characterized by the uncontrolled proliferation and dissemination of abnormal cells. If this dissemination progresses unchecked to the stage known as metastasis, it can ultimately result in mortality. The etiology of cancer involves many external factors, such as chemical exposure, radiation, and infectious agents, and internal factors, including inherited genetic mutations, hormonal imbalances, immune system dysfunctions, and stochastic genetic alterations.^[^ ^1^, ^2^ ^]^ Breast cancer is one of the most commonly diagnosed cancers worldwide and remains a leading cause of cancer‐related mortality.^[^ ^3^ ^]^ The most common treatment methods for breast cancer include chemotherapy, surgery, and radiotherapy. However, these approaches are often associated with various adverse effects, which can complicate the treatment process.^[^ ^4^ ^]^ Furthermore, the presence of metastasis and drug resistance, which are also observed in other types of cancer, presents additional challenges to the effective treatment of the disease.^[^ ^5^, ^6^ ^]^ Consequently, developing novel therapeutic strategies with minimal side effects is essential.^[^ ^7^, ^8^ ^]^ Medicinal and aromatic plants have been employed for therapeutic purposes and in cosmetic, pharmaceutical, and food industries throughout human history because of their bioactive components.^[^ ^9^, ^10^, ^11^ ^]^ The existing literature indicates that plants can be utilized as alternative therapeutic agents for cancer treatment.^[^ ^12^, ^13^, ^14^ ^]^ Turkey is located in the Northern Hemisphere and is a convergence point for three distinct phytogeographical regions: the Euro‐Siberian, Irano‐Turanian, and Mediterranean zones. The country's geopolitical position contributes to its plant diversity. *Stachys* species, which are medicinal and aromatic plants, belong to the Lamiaceae family.^[^ ^15^ ^]^ In the flora of Turkey, *Stachys* was revised by Bhattacharjee (1982). The endemic taxa are mostly Eastern Mediterranean elements. Turkey, as a center of *Stachys* diversity, contains about 91 species,^[^ ^16^ ^]^ including *S. longiflora* Boiss. & Bal. (subsect. Fragiles), a local endemic species. *Stachys* species extracts have been employed in various treatments due to their wound‐healing, antipyretic, menstrual cycle‐regulating, cardiotonic, diuretic, antidiarrheal, and anti‐inflammatory properties, including the prevention of kidney and other organ inflammations.^[^ ^17^, ^18^ ^]^ Additionally, studies have demonstrated that some *Stachys* species can reduce oxidative stress and hyperglycemia.^[^ ^17^ ^]^ The bioactive compounds present in *Stachys* species possess antimicrobial, anti‐inflammatory, antioxidant, antiviral, anticancer, and sedative properties.^[^ ^17^, ^19^, ^20^, ^21^, ^22^ ^]^

Treating infectious diseases is becoming increasingly challenging with the rising prevalence of antibiotic‐resistant microorganisms. Notably, biofilm‐producing microorganisms are central to this resistance. A biofilm is a structured community of microorganisms embedded in a self‐produced polymeric matrix that adheres to a surface. Many environmental factors influence biofilm formation, including nutrient availability, bacterial strain, pH value, temperature, and substrate surface properties.^[^ ^23^ ^]^ The extracellular matrix, which comprises proteins and exopolysaccharides, and the adhesion surface are crucial in biofilm formation. They provide a protective environment that shields the embedded bacteria from external conditions. Several studies have indicated that plants’ therapeutic effects rely on the synergistic interactions of multiple components rather than a single component. These interactions may enhance treatment efficacy by counteracting the resistance mechanisms of microorganisms that are difficult to eradicate with a single antibiotic.^[^ ^24^ ^]^ This observation has prompted researchers to investigate the inhibitory compositions of natural antimicrobial agents derived from plant extracts.

Here, for the first time, the anticancer activity of endemic *S. longiflora* (SL) extract was evaluated on the MCF‐7 cell line, and its anti‐inflammatory and antibiofilm effects were determined on *Staphylococcus aureus* ATCC 25923, known for its biofilm‐forming ability.

## 2 Results

Thirty phytochemicals were tested by LC‐MS/MS to determine the content of the SL extract. The study identified 19 phytochemicals in the SL extract, with verbascoside (12 290 ± 21 μg g^−1^ extract) and chlorogenic acid (8 872 ± 11 μg g^−1^ extract) being the major components (**Table** **1**).

**Table 1:** Phytochemical profiles of the SL extract.

No	Analyte	Concentration [μg g^−1^ extract]	No	Analyte	Concentration [μg g^−1^ extract[
1	Chlorogenic acid	8 872 ± 11	16	Luteolin	0.93 ± 0.07
2	Verbascoside	12 290 ± 21	17	Pyrocatechol	1.23 ± 0.08
3	Protocatechuic acid	116 ± 1	18	2,5‐Dihydroxybenzoic acid	0.74 ± 0.01
4	Apigenin 7‐glucoside	82.7 ± 1.5	19	3,4‐Dihydroxyphenylacetic acid	1.99 ± 0.04
5	Syringic acid	105 ± 6	20	(+)‐Catechin	nda)
6	Caffeic acid	75.0 ± 0.2	21	(–)‐Epicatechin	nd
7	Ferulic acid	53.7 ± 1.0	22	3‐Hydroxybenzoic acid	nd
8	4‐Hydroxybenzoic acid	66.1 ± 0.7	23	Taxifolin	nd
9	*p*‐Coumaric acid	62.8 ± 0.2	24	Hesperidin	nd
10	Luteolin 7‐glucoside	20.0 ± 0.2	25	Rosmarinic acid	nd
11	Hyperoside	7.3 ± 0.5	26	2‐Hydrocinnamic acid	nd
12	Gallic acid	15.3 ± 0.2	27	Pinoresinol	nd
13	Apigenin	7.6 ± 0.2	28	Eriodictyol	nd
14	Vanillin	12.6 ± 0.6	29	Quercetin	nd
15	Sinapic acid	4.3 ± 0.4	30	Kaempferol	nd

a) nd = not detected.

**Figure 1:**
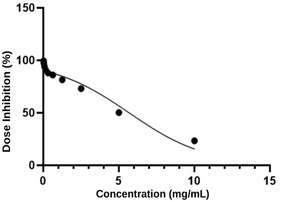
In vitro cytotoxicity of SL extract on MCF‐7 cell line.

The IC_50_ value of the SL extract on the MCF‐7 breast cancer cell line was determined as 5 mg mL^−1^ (**Figure** **1**).

The TAS, TOS, and oxidative stress index (OSI) values of the cell lysates are presented in **Table** **2**. The highest TAS value was detected in the ½ × IC_50_ group as 3.73 ± 0.01. The highest TOS and OSI values were determined in the 2 × IC_50_ group as 35.96 ± 0.75 and 19.22, respectively.

**Table 2:** TAS, TOS, and OSI values of the cell lysates.

Experimental groups	TAS [mmol Trolox equiv./L]	TOS [μmol H_2_O_2_ equiv./L]	OSI
Control	0.78 ± 0.05c)	3.28 ± 0.06a)	4.20
2 × IC_50_	1.87 ± 0.03b)	35.96 ± 0.75b)	19.22
IC_50_	1.82 ± 0.05b)	34.73 ± 1.25b)	19.08
½ × IC_50_	3.73 ± 0.01a)	19.21 ± 0.98c)	5.15

a–c) Different lowercase letters indicate statistically significant differences between groups (*p* < 0.05, ANOVA followed by Tukey's post hoc test).

**Figure 2:**
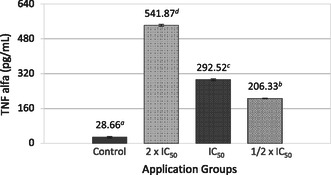
Effect of SL extract on TNF‐α cytokine level of MCF‐7 cells. Different lowercase letters (a–d) indicate statistically significant differences between groups (*p* < 0.05, ANOVA followed by Tukey's post hoc test).

The impact of the SL extract on the production of TNF‐α, TGF‐β, IL‐1β, and DEF‐β cytokines in MCF‐7 cells was investigated. The highest TNF‐α levels (541.87 ± 4.75) in MCF‐7 cells were observed at the 2 × IC_50_ SL extract application dose compared with untreated cells (**Figure** **2**).

**Figure 3:**
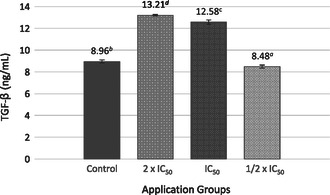
TGF‐β cytokine levels in response to treatment with SL extract compared to untreated cells (‐) in MCF‐7 cell line. Different lowercase letters (a–d) indicate statistically significant differences between groups (*p* < 0.05, ANOVA followed by Tukey's post hoc test).

The highest TGF‐β cytokine value was determined as 13.21 ± 0.08 ng mL^−1^ in the 2 × IC_50_ application group (**Figure** **3**).

**Figure 4:**
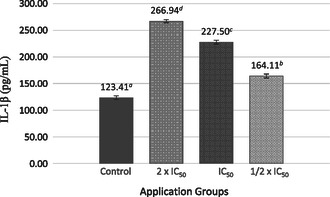
IL‐1β cytokine levels in response to treatment with SL extract compared to untreated cells (‐) in MCF‐7 cell line. Different lowercase letters (a–d) indicate statistically significant differences between groups (*p* < 0.05, ANOVA followed by Tukey's post hoc test).

IL‐1β cytokine levels were compared with the control group. The highest level was 266.94 ± 3.15 pg mL^−1^ at the 2 × IC_50_ dose. IL‐1β levels increased in a dose‐dependent manner with increasing SL extract concentrations (**Figure** **4**).

**Figure 5:**
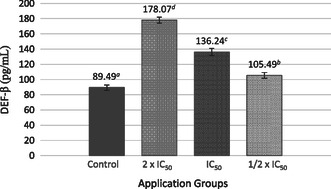
Effect of SL extract on DEF‐β cytokine level of MCF‐7 cells. Different lowercase letters (a–d) indicate statistically significant differences between groups (*p* < 0.05, ANOVA followed by Tukey's post hoc test).

When the impact of the SL extract on DEF‐β cytokine levels in MCF‐7 cells was evaluated against the control group, a dose‐dependent increase in cytokine levels was observed. The highest DEF‐β level was 178.07 ± 3.85 pg mL^−1^ in the group treated with 2 × IC_50_ SL extract (**Figure** **5**).

**Figure 6:**
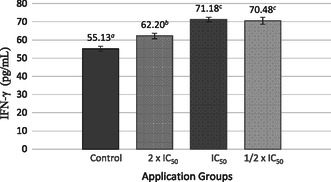
IFN‐γ cytokine levels in response to treatment with SL extract compared to untreated cells (‐) in MCF‐7 cell line. Different lowercase letters (a–c) indicate statistically significant differences between groups (*p* < 0.05, ANOVA followed by Tukey's post hoc test).

IFN‐γ cytokine levels in MCF‐7 cells increased in the SL extract‐treated groups compared with the control group. The highest IFN‐γ level was observed at the IC_50_ dose, with 71.18 ± 1.25 pg mL^−1^ (**Figure** **6**).

**Figure 7:**
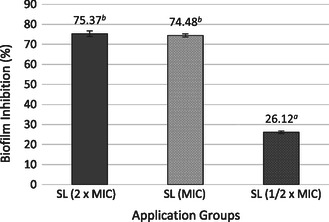
Effect of plant extract on biofilm inhibition of *S. aureus* ATCC 25923. Different lowercase letters (a,b) indicate statistically significant differences between groups (*p* < 0.05, ANOVA followed by Tukey's post hoc test).

The MIC of the *S. longiflora* extract against *S. aureus* was determined to be 250 μg mL^−1^. The MTT reduction test was employed to ascertain the antibiofilm effect of ½ × MIC, MIC, and 2 × MIC concentrations of the SL extract against *S. aureus* ATCC 25923. The biofilm inhibition increased with the concentration of the SL extract. The results revealing the antibiofilm effect of different SL extract concentrations on *S. aureus* ATCC 25923 are presented in **Figure** **7**. Biofilm inhibition was measured at 74.37% ± 1.39% in the group treated with the SL extract at 2 × MIC concentration. In the group treated with the SL extract at the MIC concentration, the inhibition was 74.48% ± 0.88%, while in the group treated with the SL extract at ½ × MIC concentration, it was 26.12% ± 0.62%.

**Figure 8:**
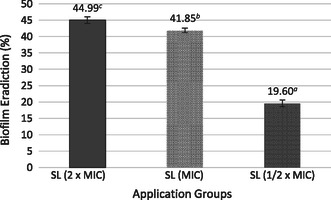
Effect of SL extract on biofilm eradiction against *S. aureus* ATCC 25923. Different lowercase letters (a–c) indicate statistically significant differences between groups (*p* < 0.05, ANOVA followed by Tukey's post hoc test).

The biofilm eradication of ½ × MIC, MIC, and 2 × MIC concentrations of the plant extract on the biofilm structure formed by *S. aureus* ATCC 25923 was determined (**Figure** **8**). The percentage of biofilm eradication also increased with the SL concentration. At 2 × MIC, biofilm eradication was 44.99% ± 1.03% in the SL extract‐treated group; at MIC, it was 41.84% ± 0.65%; and at ½ × MIC, it was 19.60% ± 0,97%.

**Figure 9:**
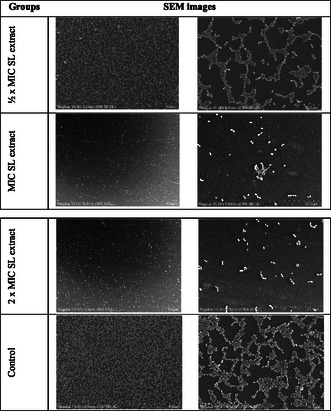
Effect of SL extract on *S. aureus* ATCC 25923 biofilm formation.

The antibiofilm effect of the SL extract on *S. aureus* ATCC 25923 was determined by scanning electron microscopy (SEM) (**Figure** **9**). A decrease in biofilm structure and cell number was observed in the ½ × MIC SL extract group compared with the control group. Structural degradation and lysis were observed in the cells. A significant decrease in cell number was observed in the group treated with MIC compared with the control group. Morphological deterioration, cell fusion, and lysis of cells were observed. Biofilm formation and cell number were significantly reduced in the 2 × MIC SL extract group compared with the control group. The morphological structures of the cells were disrupted, cell fusions occurred, and the cells were lysed. The SEM analysis results revealed that the SL extract had a significant antibiofilm activity against *S. aureus* ATCC 25923.

## 3 Discussion

In this study, the phytochemical content, anti‐inflammatory, anticarcinogenic, and antibiofilm effects of the endemic *S. longiflora*, a plant of medical importance, have been revealed for the first time. Nineteen different phytochemicals were detected in the SL extract through LC‐MS/MS analysis. Verbascoside (12 290 ± 21 μg g^−1^) and chlorogenic acid (8 872 ± 11 μg g^−1^) were identified as the major components.

Verbascoside is a distinctive secondary metabolite characteristic of the genus *Stachys*. Verbascoside and leucoseptoside A have been identified in the following *Stachys* species: *S. officinalis*, *S. recta, S. affinis*, *S. alpina* subsp. dinarica, *S. anisochilus*, *S. beckiana*, *S. byzantine*, *S. candida*, *S. schtschegleevii*, and *S.* *thracica*.^[^ ^21^, ^25^, ^26^ ^]^ Both compounds possess a wide range of biological activities, such as antioxidant, anti‐inflammatory, hepatoprotective, and antidiabetic properties^[^ ^27^, ^28^ ^]^ demonstrated that the primary compound present in various *Stachys* species was verbascoside. In another study,^[^ ^29^ ^]^ observed that *S. cretica* subsp. vacillans exhibited the highest verbascoside concentration, followed by chlorogenic acid. It was demonstrated to possess a multitude of biological activities, including radical scavenging capacity and antitumor, antimicrobial, anti‐inflammatory, and antithrombotic properties.^[^ ^30^ ^]^ Additionally, the antimicrobial activity of verbascoside against *S. aureus* was reported to occur through leucine uptake inhibition, suggesting that its mechanism may also be membrane‐related.^[^ ^31^ ^]^

The NMR spectra of *S. scardica* extract revealed the prominence of chlorogenic acid, underscoring its significance. This biologically active phenolic acid is a distinctive characteristic of the Asteraceae and Lamiaceae plant families.^[^ ^32^ ^]^ Chlorogenic acid is renowned for its diverse therapeutic attributes, which include antioxidant, antibacterial, hepatoprotective, cardioprotective, anti‐inflammatory, antipyretic, neuroprotective, and antiviral properties. Studies have indicated its potential to influence lipid metabolism and glucose levels, offering promise for addressing genetic and acquired metabolic disorders.^[^ ^32^ ^]^ Carev and Sarikurkcu (2021) observed that the quantity of chlorogenic acid present in the methanol extracts of various *Stachys* species ranged from 95.55 to 35 372 μg g^−1^, while in the aqueous extracts, this range extended from 426.30 to 17 772 μg g^−1^.^[^ ^28^ ^]^

Furthermore, studies have demonstrated that the primary constituents of *S. setifera* were terpenoids and flavonoids. Additionally, eugenol represents the primary compound of the essential oil derived from this plant. The presence of eugenol in the chloroform extract of this plant is postulated to be the reason why eugenol inhibits breast cancer.^[^ ^33^, ^34^, ^35^, ^36^ ^]^ The phenolic acid components identified in *Stachys* species include caffeic acid,^[^ ^37^ ^]^ chlorogenic acid,^[^ ^38^ ^]^ and 4‐hydroxybenzoic acid.^[^ ^38^ ^]^ Some flavonoids and flavonol glycosides include apigenin,^[^ ^39^ ^]^ casticin,^[^ ^40^ ^]^ and kumatakenin.^[^ ^41^ ^]^ Some phenylethanoid glycosides include verbascoside^[^ ^42^ ^]^ and martinoside.^[^ ^43^ ^]^

The cytotoxic effect of the SL extract on the MCF‐7 cell line was determined using the MTT test, and the IC_50_ value was determined as 5 mg mL^−1^. Similar to our study, Lachowicz‐Wiśniewska et al. (2022) studied the antiproliferative activities of the leaves, flowers, stem, and roots of *S. palustris* L. on A549 (lung cancer), BxPC3 (pancreatic ductal cancer), HT‐29 (colon cancer), CACO‐2 (colorectal cancer), HCV29T (breast cancer), and AML‐NEV007 (acute myeloid leukemia) cell lines. Their findings indicated that the leaf extract of *S. palustris* L. exhibited a notable reduction in metabolic activity across all tested cell lines with a maximum observed decrease of 8–22%. The inhibitory effect of the flower extract was observed to be comparatively weaker, with residual activity levels recorded between 10 and 52%. The root and stem extracts demonstrated the least pronounced effect, reducing cell viability by 30–90%. In another study,^[^ ^20^ ^]^ the cytotoxicity of several *Stachys* species was investigated on A549, HeLa, and MCF‐7 cells. The stem extract of *S. recta* and *S. palustris* exhibited anticarcinogenic effects on HeLa cells.

In this study, treatment of MCF‐7 breast cancer cells with SL extract led to a significant, dose‐dependent increase in several key cytokines, including TNF‐α, TGF‐β, IL‐1β, DEF‐β, and IFN‐γ. These signaling molecules regulate cell survival, growth, differentiation, and effector functions, serving as mediators of communication in multicellular organisms.^[^ ^44^ ^]^ Beyond these physiological roles, cytokines can also be secreted by cancer cells, where they may either inhibit or promote tumor growth.^[^ ^45^, ^46^, ^47^ ^]^ As part of the host defense system, the immune system plays a crucial role in modulating cancer progression, largely through the production of cytokines and other cellular mediators. Understanding the dynamic actions of cytokines during tumor development may help to elucidate the complex interplay between the immune system and cancer, offering valuable insights into disease mechanisms and potentially guiding the development of novel therapeutic strategies.^[^ ^48^ ^]^

The highest TNF‐α levels were observed at the 2 × IC_50_ dose, indicating a robust pro‐inflammatory response. Higher TNF‐α levels were observed in invasive carcinomas compared to benign tissues, with their expression increasing alongside the tumor grade. In malignant breast biopsies, TNF‐α was abundant and primarily secreted by TAM “hotspots,” targeting tumor and endothelial cells. Although TNF‐α does not directly influence angiogenesis, its receptor‐bound form on tumor cell surfaces correlates with lymph node status, suggesting a role in metastatic potential.^[^ ^49^ ^]^ Recent studies revealed minimal TNF‐α expression in normal breast epithelial cells but significantly elevated levels in tumor cells, particularly in invasive ductal carcinomas with relapse compared with ductal in situ carcinoma.^[^ ^50^ ^]^ Additionally, TNF‐α concentrations were higher in the sera of breast cancer patients than in healthy individuals, with serum levels distinguishing benign from malignant cases.^[^ ^51^, ^52^ ^]^ TNF‐α also regulates apoptosis and proliferation in breast cancer cell lines, although the mechanisms balancing these opposing effects remain unclear. This duality is influenced by the cellular context and the tumor's specific molecular characteristics.^[^ ^53^ ^]^

The elevated TGF‐β levels following SL extract treatment further highlight the complexity of cytokine signaling in cancer. The TGF‐β signaling pathway plays a pivotal role in tumorigenesis and is frequently altered during tumor progression in human cancers. According to current literature and experimental findings, TGF‐β functions as a potent ligand that regulates cancer initiation, progression, and metastasis through a wide and intricate network of interdependent interactions. Genetic alterations in the TGF‐β pathway often involve mutations, deletions, or amplifications of TGF‐β ligands and their receptors.^[^ ^54^ ^]^ High TGF‐β levels are produced by various tumors, including melanomas and breast, colon, esophagus, stomach, liver, lung, pancreas, and prostate cancers, as well as hematologic malignancies.^[^ ^55^, ^56^ ^]^ In early tumorigenesis, TGF‐β acts as a tumor suppressor. However, gastrointestinal tumors with microsatellite instabilities or TGF‐βRII mutations and pancreatic cancers with Smad4 mutations resist its signaling and antitumor effects.^[^ ^57^, ^58^ ^]^ In later stages, TGF‐β promotes tumor growth, progression, and metastasis by facilitating the epithelial‐to‐mesenchymal transition and tumor angiogenesis.^[^ ^59^, ^60^ ^]^ Notably, metastatic breast and colon tumors exhibit higher TGF‐β expression than their primary counterparts, highlighting its role in advancing tumor progression.^[^ ^61^, ^62^ ^]^

Human beta defensin‐1 is a powerful “microchemokine” that attracts immature dendritic cells and memory T cells by interacting with the chemokine receptor CCR6, which plays a key role in innate and adaptive immunity. However, its role in prostate tumor progression remains uncertain.^[^ ^63^ ^]^ The observed upregulation of DEF‐β in this study suggests a potential impact on innate immune responses; nevertheless, its specific role in breast cancer progression has yet to be fully elucidated. Based on the results obtained, DEF‐β may contribute to modulating the tumor microenvironment, although further research is necessary to clarify its precise function.

In the present study, IL‐1β also exhibited a dose‐dependent increase, consistent with its established role in promoting tumor invasiveness and aggressive behavior through activation of signaling pathways. IL‐1β plays a dual role in cancer, promoting adaptive antitumor responses during acute inflammation while increasing cancer risk under chronic inflammatory conditions.^[^ ^64^ ^]^ In human breast cancer, elevated IL‐1β expression is linked to tumor invasiveness and aggressive behavior.^[^ ^65^ ^]^ The expression of IL‐1α, IL‐1β, and their receptors in breast cancer tissues activates cells in the tumor microenvironment, contributing to angiogenesis, proliferation, and invasion.^[^ ^66^ ^]^ IL‐1β also induces IL‐6 production in TG2‐expressing MCF7 cells via NF‐kB‐, PI3K‐, and JNK‐dependent pathways, enhancing stem‐like traits, invasiveness, and estrogen‐independent tumor growth. These effects are mitigated by anti‐IL‐6 or anti‐IL‐1β antibody treatment.^[^ ^67^ ^]^

IFN‐γ levels were primarily elevated at the IC_50_ dose, underscoring its significant immunoregulatory and anti‐tumor functions, which likely play a critical role in balancing tumor suppression and inflammation‐mediated tumor promotion. IFN‐γ is a prototypical multifunctional and pleiotropic cytokine. Beyond its involvement in fundamental physiological processes, it plays a central role in immune cell function and in regulating both innate and adaptive immune responses, particularly within the context of tumor immunoediting.^[^ ^68^ ^]^ The biological effects of IFN‐γ are well documented and include enhanced pathogen recognition, increased antigen processing and presentation, inhibition of cell proliferation, regulation of apoptosis, activation of antimicrobial mechanisms, modulation of immune responses, facilitation of leukocyte trafficking, and induction of cytostatic and antitumor activities during adaptive immune responses.^[^ ^69^ ^]^

The results of the present study underscore the complex and multifactorial roles of cytokines in the pathophysiology of breast cancer and suggest that SL extract may exert its effects, at least in part, by modulating the tumor microenvironment through cytokine regulation. Given the pleiotropic and sometimes contradictory functions of these molecules, further in‐depth mechanistic and functional studies are warranted to elucidate the precise biological effects of SL extract. Moreover, the observed alterations in cytokine profiles may serve as a basis for the identification of novel biomarkers or therapeutic targets, particularly in inflammation‐associated tumor progression. Despite the valuable insights provided by the present study, several limitations should be acknowledged. As this research was conducted under in vitro conditions, the results may not fully reflect the complex interactions that occur in vivo. Additionally, while the study focused on cytokine levels and their association with breast cancer, functional experiments to establish direct causal relationships were not performed. Patient‐specific factors such as genetic background, hormonal status, and clinical stage were also not controlled for, which may influence cytokine expression profiles. Therefore, future studies incorporating larger cohorts, in vivo models, and detailed mechanistic analyses are essential to validate and expand upon these findings.

Oxidative stress arises when the body's antioxidative mechanisms fail to neutralize active oxidants produced by harmful stimuli.^[^ ^70^ ^]^ Reactive oxygen species (ROS), comprising over 95% of active oxides, are critical in cancer development; they induce DNA damage and genetic mutations, inhibit apoptosis, and promote malignant cell proliferation, invasion, and metastasis.^[^ ^71^ ^]^ Oxidative stress is characterized by an imbalance between oxidants and antioxidants, necessitating the measurement of TOS and TAS to evaluate the body's overall oxidative state.^[^ ^72^, ^73^ ^]^ The OSI, calculated as the TOS/TAS ratio, offers a more precise measure of oxidative stress.^[^ ^74^ ^]^ Oxidative stress has been implicated in breast cancer pathogenesis, where an imbalance between ROS levels and oxidative defenses leads to biomolecular damage, cellular alterations, and tumorigenesis.^[^ ^75^, ^76^ ^]^ Excessive ROS generated by various risk factors can disrupt cellular homeostasis, contributing to neoplastic transformation.^[^ ^4^, ^77^ ^]^

A biofilm is a structured community of microorganisms embedded in a self‐produced polymeric matrix that adheres to the surface. Its formation is influenced by various environmental factors, such as nutrient availability, bacterial strain, pH, temperature, and substrate surface properties.^[^ ^23^ ^]^ The present study demonstrated that the SL extract exerted an antibiofilm effect on *S. aureus* at 2 × MIC SL, with 74.37% ± 1.39% inhibition of biofilm formation and 44.99% ± 1.03% of biofilm eradication,^[^ ^78^ ^]^ revealing that the phenolic compounds have a significant antibiofilm activity. Apigenin, a phenolic compound, inhibits *Streptococcus sobrinus* biofilm formation.^[^ ^79^ ^]^ Gallic acid at 4 mg mL^−1^ reduced the formation of *S. aureus* biofilm by ≈40% and affected the metabolic activity of adherent cells, even at low concentrations. It caused bacterial cell wall damage and deformation, surpassing the effects observed in the positive control group. Similarly, verbascoside, used as an antibacterial additive in fresh meat storage, effectively killed bacteria at medium and high doses, reducing spoilage rates and prolonging meat shelf life.^[^ ^80^, ^81^ ^]^ Chlorogenic acid significantly inhibited biofilm formation by *Yersinia enterocolitica* in a dose‐dependent manner, reducing the biofilm layer effectively.^[^ ^81^ ^]^

In recent years, the antimicrobial potential of some *Stachys* species has been of great interest to various research groups. Researchers revealed that various polar extracts, as well as essential oils, have antimicrobial activities against human pathogens, including *S. aureus* and *Pseudomonas aeruginosa*.^[^ ^82^, ^83^, ^84^ ^]^ Napolitano et al. (2022) revealed that the *S. spreitzenhoferi* extract could inhibit the growth of gram‐positive bacteria, affecting the cell wall of the microorganism.^[^ ^85^ ^]^ Saeedi et al. (2008) evaluated the use of *S. byzantina*, *S. inflata*, *S. lavandulifolia*, and *S. laxa* extracts against *S. aureus*, *Streptococcus sanguis*, *Escherichia coli*, *Pseudomonas aeruginosa*, *Klebsiella pneumoniae*, *Aspergillus niger*, and *Candida albicans*. They found that the plant extracts exhibited antibacterial activity against the bacteria tested, with stronger effects observed against gram‐positive microorganisms.^[^ ^86^ ^]^

## 4 Conclusions

Natural products are increasingly being considered as alternatives to commonly used chemicals in antimicrobial treatments. Our study investigated the phytochemical composition and antimicrobial, antioxidant, antibiofilm, anti‐inflammatory, and antitumor activities of SL extract. Notably, the methanol extract of this plant contains significant amounts of verbascoside and chlorogenic acid, which may contribute to its potent antioxidant properties. It is important to recognize that cytokines play a dual role in human health, exerting beneficial and adverse effects. Therefore, a better understanding of the cytokines involved in the response induced by this plant is essential to optimize its use in anticancer therapies. The variable efficacy of cytokine‐based immunotherapies highlights the need for further research. Additionally, the high antibiofilm activity demonstrated by SL extract highlights its potential in treating biofilm‐associated infections. These findings highlight the potential of SL as a promising source of bioactive compounds with antibiofilm and anticancer properties. Further studies are required to investigate how other signaling pathways related to the cell cycle and apoptosis are affected, in healthy cells and other cancer cell lines, as well as in in vivo models. Additionally, further research focusing on the isolation and purification of the compounds in the SL extract may help identify its full potential as a source of natural anticancer agents.

## 5 Experimental Section

5.1

5.1.1

##### Plant Material

The SL plants used in this study were collected from the Mersin Province in May 2023 and transported to the laboratory. Dr. Olcay CEYLAN, from the Biology Department at Muğla Sıtkı Koçman University, confirmed the species identity and preserved it in the herbarium with the accession number O.2235. The samples were initially dried and ground in an environment free from direct sunlight, with a low humidity level and adequate air circulation. An ultrasonic extraction method was employed to prepare the SL methanol extract. 30 g of powdered sample was subjected to an hour‐long extraction by ultrasonication in 400 mL of solvent (methanol:distilled water 1:1) at 37 °C. The extracts were then filtered through a Whatman No. 1 filter paper. Throughout the protocol, the temperature was maintained below 40 °C to prevent compound degradation. The solvents were then completely evaporated and lyophilized in a rotary evaporator (Heidolph) at low pressure. The SL extract was stored at 4 °C^[^ ^87^ ^]^ until further use.

##### Liquid Chromatography‐Electrospray Tandem Mass Spectrometry Analysis

The phytochemicals in the extracts were analyzed by liquid chromatography‐electrospray tandem mass spectrometry (LC‐ESI‐MS/MS). An Agilent Technologies 1260 Infinity LC system coupled with a 6420 Triple Quad mass spectrometer was used. Chromatographic separation was conducted on a Poroshell 120 EC‐C18 column (100 × 4.6 mm I.D., 2.7 μm) under previously reported analytical conditions.^[^ ^88^ ^]^

##### Anticancer Activity of the Plant Extract

The 3‐(4,5‐dimethylthiazol‐2‐yl)‐2,5‐diphenyltetrazolium bromide (MTT) test was used to determine the cytotoxic effect of the SL extract on MCF‐7 cells.^[^ ^89^ ^]^ MCF‐7 cells were incubated in Dulbecco's modified Eagle's medium containing 10% fetal bovine serum and 1% penicillin–streptomycin (Thermo‐Fisher) and incubated in a humidified atmosphere of 5% CO_2_ at 37 °C for 24 h. MCF‐7 breast cancer cells were first seeded in 96‐well plates at 2 × 10^4^ cells per well. After reaching the 70–80% confluency, cells were treated with twofold increasing concentrations of SL extract (0.019–10 mg mL^−1^) and incubated in the same conditions for 24 h. Next, the MTT reagent was added to each well, and plates were incubated at 37 °C for 2 h. After incubation, the MTT reagent was removed, and dimethyl sulfoxide was added to each well. The plate was then shaken at low speed for 5 min at room temperature. The absorbance values were quantified using an ELISA reader (Multiscan Sky, Thermo Fisher Scientific) at 570 nm, and the cell viability was calculated. The untreated cells served as the control group and were considered to be 100% viable. The results were expressed as the percentage of treated cells relative to the controls. All experiments were performed in triplicate, and the IC_50_ values were determined using GraphPad Prism 9 (GraphPad Software, Inc., USA).

##### Preparation of the Cell Lysate

Four treatment groups were applied to MCF‐7 breast cancer cells at a confluency of 50–60%: one control group (medium) and three treatment groups with different concentrations of SL extract (½ × IC_50_, IC_50_, and 2 × IC_50_). The cells were incubated in a humidified atmosphere of 5% CO_2_ at 37 °C for 24 h. After incubation, the cells that had adhered to the flask bottom were removed through trypsinization and trypsinization procedures. Subsequently, the cells were washed twice with the culture medium to remove any residual trypsin. The residual cell pellet at the base of the Falcon tube was washed twice with ice‐cold phosphate‐buffered saline (PBS) and removed by scraping. The cells were then lysed in lysis buffer (100 mM NaH_2_PO_4_, 1% Triton X‐100, 1 M HEPES, and 1% protease inhibitor cocktail) and subsequently centrifuged at 13 000 rpm at 4 °C for 40 min.^[^ ^90^ ^]^

##### Total Oxidant Status, Antioxidant Status, and Oxidative Stress Index

The protein concentrations of the cell lysates were determined using the Bradford Protein Assay Kit (Quick Start, BIO‐RAD). The total oxidant status (TOS) and antioxidant status (TAS) of the cell lysate were measured by automated colorimetric methods following the manufacturer's protocol (Elabscience Biotechnology Inc). The results were expressed as μmol H_2_O_2_ equiv./L and mmol Trolox equiv./L for TOS and TAS, respectively.

##### Effect of the Plant Extract on Cytokine Production

Inflammatory cytokine levels (TNF‐α, DEF‐β, IL‐1β, TGF‐β, and IFN‐γ) in the cell lysate were analyzed by ELISA technique. The cytokine concentrations in 100 μL of each cell supernatant were determined according to the manufacturer's protocol (Wuhan USCN Business Co., Ltd). The results are expressed as ng mL^−1^ for TGF‐β and pg mL^−1^ for the other cytokines. All incubation steps were conducted at room temperature. The optical density at 450 nm was measured with an ELISA reader.

##### Evaluation of the In Vitro Antimicrobial Activity

The minimum inhibitory concentration (MIC) value of the SL extract was determined using the Clinical Laboratory Standards Institute (CLSI) M7‐A8 protocol.^[^ ^91^, ^92^ ^]^ The broth microdilution test was conducted using sterile, U‐bottom, and 96‐well microplates. *S. aureus* ATCC 25923 was incubated at 37 °C for 24 h in a Müller–Hinton broth medium. 100 μL of the bacterial suspension (1 × 10^6^ CFU mL^−1^) was added to the wells. Subsequently, 100 μL of twofold serial concentrations of SL extract (0.244–1000 μg mL^−1^) were added to each well and incubated overnight at 37 °C. After incubation, the absorbance was measured at 545 nm with an ELISA reader. 100 μL of a ciprofloxacin solution (1 mg mL^−1^) was employed as a positive control, while the medium served as a negative control.^[^ ^93^ ^]^ The lowest concentration at which no microbial growth was observed was identified as the MIC value in comparison with the control groups. All analyses were conducted in triplicate.

##### Antibiofilm Effects against S. aureus ATCC 25923

The MTT reduction test was employed to determine the antibiofilm impact at high, medium, and low concentrations (½ × MIC, MIC, and 2 × MIC concentrations) of the SL extract.^[^ ^94^ ^]^ *S. aureus* ATCC 25923 was incubated in a Triptic Soy Broth medium containing 1% glucose for 24 h at 37 °C. 100 μL of bacterial suspension at 1 × 10^6^ CPU mL^−1^ was added to a 96‐well plate. Subsequently, 100 μL of each treatment group was added to the 96‐well plates and incubated at 37 °C for 24 h. 100 μL of ciprofloxacin (1 mg mL^−1^) was used as the positive control, and the medium was used as the negative control. After incubation, the supernatants were removed, and the wells were washed three times with PBS. 150 μL of PBS and 50 μL of MTT (0.3%) were added to the wells and incubated at 37 °C for 2 h. The MTT solution was then removed from the wells, and 150 μL of dimethyl sulfoxide and 25 μL of 0.1 M glycine buffer (pH 10.2) were added to the wells to facilitate the dissolution of the formazan crystals. The plates were then incubated for 15 min at room temperature. Subsequently, the optical density was determined at 570 nm using an ELISA reader. The inhibition percentage was calculated for each treatment group using the following equation. All analyses were conducted in triplicate.

(1) $$\text{The percentage inhibition }(\%)=\left[1-\left(\frac{A_{570}\;\text{of the test}}{A_{570}\text{of untreated control}}\right)\right]\times 100$$

##### Eradication of the Biofilm Formation

The eradication of biofilm formation by the SL extract was evaluated using a minimum biofilm eradication concentration (MBEC) assay, as previously described.^[^ ^95^ ^]^ Each well of a 96‐well microtiter plate was inoculated with 200 μL of bacterial suspension (10^6^ CFU mL^−1^) and incubated for 24 h at 37 °C. After incubation, the medium was removed, and the wells were washed three times with PBS to remove nonadherent cells. 200 μL of ½ × MIC, MIC, and 2 × MIC concentrations were added to the wells and incubated for 24 h at 37 °C. The adherent bacteria were washed three times in PBS, and the numbers of surviving microorganisms were determined using an MTT assay. The MBEC value was defined as the concentration that exhibited 50% and 90% inhibition of biofilm formation on the biofilm. 100 μL of ciprofloxacin (1 mg mL^−1^) was employed as the positive control, and the medium was used as the negative control. All experiments were repeated three times. The percentage of eradication was calculated as follows

(2) $$\text{Biofilm eradication }(\%)=\left[1-\left(\frac{A_{570}\;\text{of the test}}{A_{570}\text{of untreated control}}\right)\right]\times 100$$

##### Determination of the Biofilm Structure by Scanning Electron Microscopy

The antibiofilm effect of the plant extract on *S. aureus* ATCC 25923 was determined by SEM. A previous study has determined that *S. aureus* ATCC 25923 is a strong biofilm producer.^[^ ^94^, ^96^ ^]^ For this procedure, ½ × MIC, MIC, and 2 × MIC concentrations of SL extract were added to the 24‐well flat‐bottom plate containing 14 × 14 mm polylysine slides. Then, 1,000 μL of *S. aureus* ATCC 25923 cultures (1 × 10^6^ CFU mL^−1^) were added to 24‐well microtiter plates and incubated for 24 h at 37 °C. After the application period, the incubation was stopped, and the materials were left in the primary fixative (2.5% glutaraldehyde solution prepared in PBS) for 24 h at 4 °C. The samples were washed three times with PBS for 15 min, removed from the fixative, and kept in the dark in a rotator for 1 h in the secondary fixative containing OsO_4_. Finally, they were washed again with PBS three times. The samples were dehydrated using a series of ethyl alcohol concentrations (50%, 70%, 90%, 95%, and 100%), dried using the Polaron CPD Critical Point Dryer, mounted on aluminum stubs, and coated with gold–palladium using the Polaron SC7620 Sputter Coater. Subsequently, they were imaged using an SEM (JEOL JSM‐5600LV).^[^ ^97^ ^]^

##### Statistical Analysis

Statistical analyses were performed using SPSS version 22.0 (Statistical Package for the Social Sciences) software. All results are expressed as the mean ± standard deviation (SD) of three parallel measurements (*n* = 3). Where applicable, differences between groups were evaluated using one‐way analysis of variance (ANOVA) followed by Tukey's post hoc test for multiple comparisons. A *p*‐value less than 0.05 (*p* < 0.05) was considered statistically significant.

## Conflict of Interest

The authors declare no conflict of interest.

## Author Contributions

**Ecem GÜLDAR**: conceptualization; investigation; writing—original draft; methodology; formal analysis. **Sevim Feyza ERDOĞMUŞ**: investigation; writing—review and editing; methodology; software; formal analysis; visualization. **Cengiz SARIKURKCU**: investigation; writing—review and editing; methodology; visualization; validation.

## Data Availability

The data that support the findings of this study are available on request from the corresponding author. The data are not publicly available due to privacy or ethical restrictions.
